# On Etherization

**Published:** 1847-05

**Authors:** 


*On Etherization.* Since we last noticed this subject, although the inhalation of ether has been practised to a great extent, much of the enthusiasm which at first prevailed respecting it has been dissipated. The occasional unpleasant, and in a few instances even fatal effects which have resulted from its use, have caused a salutary check to the extravagant anticipations which were formed with regard to it. Further experience only can enable us to form correct notions of those circumstances which may render its application warrantable. In the meantime it is our intention to give a short summary of the novel facts which have been elicited in connexion with etherization during the past month.

*Apparatus and mode of inhalation.*—The forms of apparatus invented for inhaling ether are already endless. The desideratum at present is to render them cheap and portable, without destroying their efficiency. The apparatus employed by Professor Simpson completely answers these purposes. (See our Report of the Med. Chir. Society, March 3d.) Experience has shown that the inhalation should be so conducted as to produce its full effect as soon as possible, in order to prevent or shorten the period of excitement. With this view a large volume of vapour should be inhaled from the first, the individual should not be disturbed, or the inhalation interrupted, and the ether should be pure.

It has been proved experimentally by Mr. Young, cutler, Edinburgh, on his own person, before the Society of Arts, that so far from there being any danger of explosion during the inhalation, that flame applied to the mouth, and breathed upon, is immediately extinguished.

*Physiological effects.*—Numerous experiments have been performed on the lower animals by MM. Flourens, Serres, Gruby, Longet, Magendie and others. The same phenomena have for the most part been observed in them as in man. Stupification by ether constitutes a convenient mode of depriving animals of sensation, for experiments on the excito-motory system. If pushed too far, however, even this is affected, and death is occasioned. Different degrees of insensibility may be produced, aud an action upon the brain proper alone, or combined with this upon the medulla oblongata, and spinal chord, be occasioned according to the extent to which inhalation is carried. According to M. Flourens, ether acts on the nervous centres in the following order :—First, on the cerebral hemisphere; second, on the cerebellum; third, one the spinal chord; lastly on the medulla oblongata, destroying successively intelligence, regular movements, sensibility, and life. Dr. Buchanan of Glasgow, having pointed out that the blood surcharged with ether is sent directly to the heart and brain, explains the evanescence of its action by comparatively pure blood from the lower regions of the body, succeeding it as soon as the inhalation is stopped. (Paper read to the Philosophical Society of Glasgow.) The peculiar sensations experienced by individuals vary considerably in different cases.

*Applications.*—The removal of pain during surgical operations still constiutes the chief object of inhalation. Even this application of it, however, has caused perhaps less sensation than that of destroying the pains of child-birth, without interfering with the progress of labour. This fact, first ascertained by Professor Simpson, of Edinburgh, has been confirmed by Professor Paul Dubois of Paris, and subsequently by several others.

We are informed by Dr. Simpson that latterly he has ascertained two important points with regard to the use of ether in midwifery. First, its action may be kept up for hours. In one case he had a patient placed for four, and in another for five hours and a half, under its influence before the child was born. When the patients awoke, about thirty or forty minutes after delivery, they were quite unconscious of the birth of their infants, and could scarcely at first be persuaded of the happy result. Both labours had been previously exceedingly tedious. One of the patients had child's head arrested at the brim, and after being above thirty-six hours in labour, was delivered by Dr. S. with the long forceps. Second; the fœtus in utero seems not to be deleteriously affected by even such prolonged etherization of the mother. In these two cases the action of the fœtal heart was carefully watched by Dr. S. with the stethoscope, and did not vary above ten or fifteen beats during the whole time of the etherization. Both children were born alive and well.

Ethereal inhalation has also been tried in several cases of facial neuralgia, inducing insensibility to the painful paroxysms, and sleep which could not otherwise be procured.

Another application has been made by M. Baudens to determine true from feigned diseases in the army. In one case, where curvature of the back was simulated, the deformity disappeared during the insensibility caused by ether, and the individual was led to confess the imposture. In another case. a suspected ankylosis of the hip joint was in the same way proved to be a reality.

*Inconvenient effects* have frequently resulted from the inhalation. Many of these will be found related by Professor Syme and Dr. Roberts, in our report of the meetings of the Medico-Chirurgical Society of Edinburgh. Great excitement, cough with expectoration of pus, hemoptysis, and convulsions, during the inhalation, have been witnessed by ourselves. In some cases, erotic feelings, and even nymphomania, have been occasioned in females, in others hysterical symptoms, or those of depression or intense headache, which have continued several days. In our last number we noticed the occasional occurrence of alarming sinking, which required vigorous measures to restore the individual. In some cases, the individuals have been thrown into such a state of agitation as to render the performance of the operation impossible.

*Fatal effects* have become multiplied. In our last number, one fatal case was noticed, occurring in the Edinburgh Royal Infirmary. We are informed that there are just now two other cases in which the ether was given, dying of secondary purulent deposits in the same institution. Is this result the effect of ether? An answer in the affirmative cannot be decidedly given, but, as we previously stated, all such cases require to be put on record. M. Jobert has brought forward two cases in which he considered death to be partly dependent on the ether. M. Roux has given another of tetanus, in which the patient never rallied from the stupefaction, and where death was decidedly accelerated by it. Mr. Nunn, of Colchester, has recorded a case of lithotomy, which sunk without the patient having rallied from the operation; and Dr. Maclagan has mentioned another, occurring in London after amputation of the thigh.

We observe in the Times, an account of an inquest at Grantham, in the County of Lincoln, in a case where an osteo-sarcomatous tumour was removed by Mr. Robbs, surgeon, under the influence of ether. The patient never rallied from the operation, which was in no way severe or prolonged, and the jury found "That the deceased, Ann Parkinson, died from the effects of the vapour of ether, inhaled by her for the purpose of alleviating pain during the removal of a tumour from her left thigh, and not from the effect of the operation, or any other cause." In the correctness of this verdict the surgeon himself, Mr. Robbs, concurred.

*Morbid Appearances.*—The morbid appearances which have been found after death, caused by ether in animals, are similar to those observed in asphyxia, namely, fluidity of the blood, its collection in the right side of the heart and large veins, and engorgement of the internal viscera. In the fatal case of the Royal Infirmary there was found double pneumonia, bronchitis, and secondary purulent deposits in the joints. In the case of Mr. Nunn, cerebral congestion, lungs engorged posteriorly, and uniform fluidity of the blood. In the case at Grantham there was no great congestion, but the blood was fluid throughout. The observations of Amussat and Lassaign have shown that in every case the blood loses its power of coagulation, although with the exception of the presence of a minute dose of ether, its chemical principles are unchanged.

*Claims to the discovery.*—The merit of discovering the application of etherization to removing pain in surgical operations, has been lately claimed by Dr. Wells, of America. He states that he was led to the discovery by observing that individuals, when in a state of great excitement, as during battle, or intoxication, never felt the pain of local injuries. He consequently caused the patient to inhale ether, and nitrous oxide gas in several cases, and found that they were thus insensible to the pain of surgical operations. He was led to prefer the nitrous oxide gas for this purpose, from its causing less injurious effects than ether. He communicated his discovery to Drs. Morton and Jackson, who then received it with incredulity. He shortly after left America for Europe, and was much surprised on arriving at Paris to find that those gentlemen had propagated his ideas without any allusion to him.

Since writing the above we have been informed that Professor Syme has abandoned the use of ether in his surgical clinic.—*Monthly Journal of Medical Science.*

